# OP54 Different Perceptions Of Additional Benefit By Payers And Providers: Discrepant Voting Within G-BA’s Benefit Appraisals

**DOI:** 10.1017/S0266462324001168

**Published:** 2025-01-07

**Authors:** Andrej Rasch, Elaine Julian, Jörg Ruof

## Abstract

**Introduction:**

Appraisal decisions on additional benefit of new medicines within the German health technology assessment (HTA) body Gemeinsamer Bundesausschuss (G-BA) are made by voting among the member of the G-BA plenary. We identified and analyzed key characteristics of decisions that were not reached by consensus.

**Methods:**

G-BA’s homepage was used to identify AMNOG (German Medicines Market Reorganization Act) procedures that started after January 2011 and were finalized before November 2023. Appraisal voting is conducted publicly, and results are documented in the data source of the German Association of Research-Based Pharmaceutical Companies (vfa). Both the payer (National Association of Statutory Health Insurance [GKV-SV]) and provider (National Association of Statutory Health Insurance Physicians, Dentists and the German Hospital Federation) “benches” have an equal number of votes with the independent chair of the G-BA acting as swing vote in case of discrepant decisions. Discrepant voting instances were extracted and analyzed.

**Results:**

From January 2011 to November 2023, G-BA conducted 908 appraisals of medicines. In 66 appraisals, (7.3%) decisions were not reached by consensus. Discrepant voting was related to oncological (n=28), metabolic (n=15), infectious (n=12), neurologic (n=3), cardiovascular (n=2), psychiatric (n=2), dermatologic (n=2), musculoskeletal (n=1) and urogenital conditions (n=1) conditions. Fourteen discrepant voting instances related to orphan medicines. The best benefit category reached in the 66 discrepant decisions were: major (n=2), considerable (n=16), minor (n=19), non-quantifiable (n=13), and no benefit (n=16). In all discrepant voting decisions, the provider bench favored a better scoring versus the payer bench.

**Conclusions:**

Appraisal decisions within G-BA are reached by voting. The appraisals are a key element within the subsequent price negotiations. In all discrepant decisions, the payer bench suggests less benefit (strength of benefit, respectively) versus the provider bench, indicating a procedural challenge with the GKV-SV being involved in both the voting on the additional benefit and the negotiation of price.

